# Structural and Mechanical Properties of Doped Tobermorite

**DOI:** 10.3390/nano13162279

**Published:** 2023-08-08

**Authors:** Xiaopeng Li, Hongping Zhang, Haifei Zhan, Youhong Tang

**Affiliations:** 1 School of Materials and Chemistry, Southwest University of Science and Technology, Mianyang 621010, China; lxp5202021@163.com; 2 School of Mechanical Engineering, Institute for Advanced Study, Chengdu University, Chengdu 610106, China; 3 College of Civil Engineering and Architecture, Zhejiang University, Hangzhou 310058, China; zhan_haifei@zju.edu.cn; 4 School of Mechanical, Medical and Process Engineering, Queensland University of Technology (QUT), Brisbane 4001, Australia; 5 Institute for NanoScale Science and Technology, College of Science and Engineering, Flinders University, Adelaide 5042, Australia

**Keywords:** calcium silicate hydrate, tobermorite, structural optimization design, doping, density functional theory, mechanical property

## Abstract

As calcium silicate hydrate (C-S-H) is the main binding phase in concrete, understanding the doping behavior of impurity elements in it is important for optimizing the structure of cementitious materials. However, most of the current studies focus on cement clinker, and the doping mechanism of impurity elements in hydrated calcium silicate is not yet fully understood. The hydrated calcium silicate component is complex, and its structure is very similar to that of the tobermorite mineral family. In this study, the effects of three different dopants (Mg, Sr and Ba) on a representing structure of C-S-H—tobermorite—was systematically explored using densify functional theory (DFT) calculations. The calculations show that Mg doping leads to a decrease in lattice volume and causes obvious structure and coordination changes of magnesium–oxygen polyhedra. This may be the reason why high formation energy is required for the Mg-doped tobermorite. Meanwhile, doping only increases the volume of the Sr- and Ba-centered oxygen polyhedra. Specifically, the Mg-doped structure exhibits higher chemical stability and shorter interatomic bonding. In addition, although Mg doping distorts the structure, the stronger chemical bonding between Mg-O atoms also improves the compressive (~1.99% on average) and shear resistance (~2.74% on average) of tobermorillonite according to the elastic modulus and has less effect on the anisotropy of the Young’s modulus. Our results suggest that Mg doping is a promising strategy for the optimized structural design of C-S-H.

## 1. Introduction

Cement is one of the most utilized building materials in the world, broadly applied in the construction of civil infrastructure, nuclear waste barriers, military bases and marine engineering [1,2,3,4,5,6,7]. Its annual usage is increasing year by year; however, the high-temperature firing process of cement is accompanied by huge industrial energy consumption and CO_2_ release [8]. After years of research, chemical doping by introducing impurity ions to adjust the microstructure of clinker minerals has been proven to be an effective method to reduce the sintering temperatures of cement [9,10,11], for example, the doping of Mg^2+^, Sr^2+^, Ba^2+^ and Al^3+^. In addition, the introduction of these impurity ions changes the local charge density and thus affects the physicochemical properties of cement-based materials, and this effect has been extensively studied [12,13,14,15,16]. Zhao et al. [17] found that Mg^2+^ dissolves into the cement clinker phase as a solid solution by replacing Ca^2+^, causing distortion in the crystal structure of clinker minerals and thus altering their properties. This substitution preference is in agreement with the findings of Zhang et al. Manzano et al. investigated the effect of impurities such as Mg^2+^, Al^3+^ and Fe^3+^ on the electronic structure of cement clinker crystals through atomic simulations and found that Mg^2+^ does not change the hydration rate, Al^3+^ slightly increases the hydration rate and Fe^3+^ causes a decrease in the hydration rate of cement [16]. Durgun et al. went further and attempted to regulate the reactivity of cement clinker using chemical substitution methods [18]. Li et al. studied the modulating effect of elements such as Ba and Zn on the hydration reactivity of β-C_2_S and proposed that Ba doping could enhance the reactivity of C_2_S [19]. Zhu et al. systematically investigated the doping mechanism of barium in sulfoaluminate cement clinker and found that Ba ions preferred to replace Ca atoms into mineral calcium sulfoaluminate [20]. In addition, the mechanical strength and seawater durability of sulphate aluminate cement can be improved in the case of BaO and SrO as admixtures [21,22].

Nevertheless, most of the current studies have focused on cement clinker minerals, and there are few reports on the mechanism of ion admixture in cement hydration products. Calcium silicate hydrate (C-S-H), as a binder for concrete, is the main product of hydration of most cements and is also the main source of the mechanical properties of cement paste [23,24,25,26]. Therefore, it is necessary to investigate the doping difficulty of impurity ions in C-S-H, the atomic structure effects, the ionic valence states, chemical bonding and the effects on the mechanical properties. These studies are important for understanding and optimizing the structure of hydrated calcium silicate, which becomes the focus of this work. However, it is often difficult to characterize these properties using experimental methods, so high-precision atomistic simulation techniques are needed to study hydrated calcium silicate gels to obtain data. In fact, this technique has become an important tool for the study of various gelling materials [7,27,28]. Based on density functional theory (DFT) calculations, the influence of three kinds of doping elements on the structure and mechanical properties of tobermorite—a representative C-S-H model—was systematically investigated [29]. It was found that Mg-doped tobermorite exhibits good chemical stability and interatomic bonding, which also possesses enhanced compressive and shear properties.

## 2. Computational Details

### 2.1. Crystal Structures and Theoretical Methods

**Figure 1:**
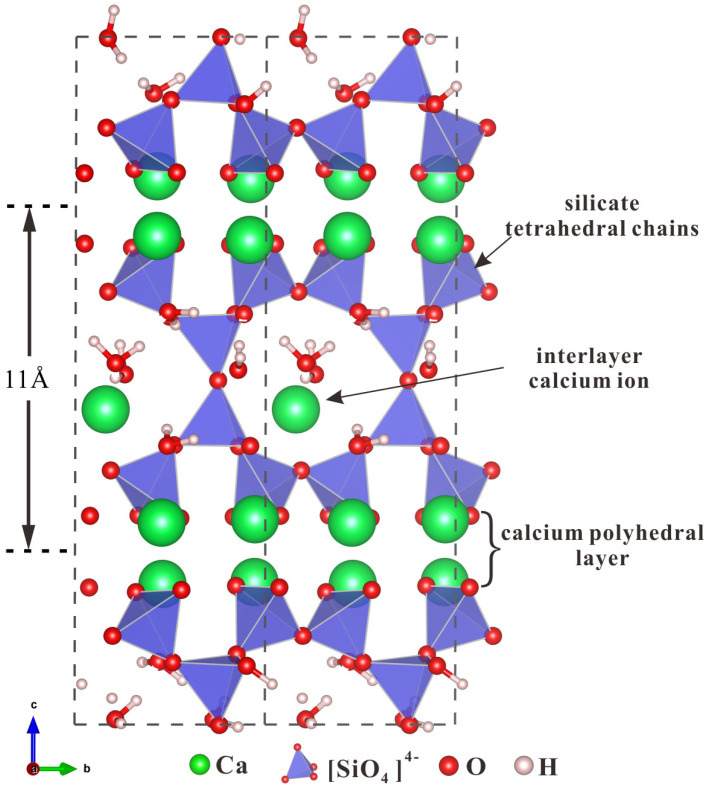
The crystal structure of relaxed tobermorite with a spacing of 11 Å. The green, red and white balls represent calcium, oxygen and hydrogen atoms, respectively. The blue block represents the silico-oxygen tetrahedron.

**Figure 2:**
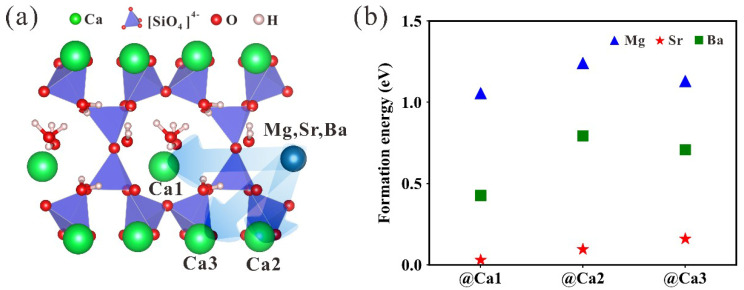
(**a**) The doping sites (Ca1, Ca2 and Ca3) and (**b**) *E_formation_* of Mg-, Sr- and Ba-doped tobermorite.

Previous studies illustrated the microstructure and chemical composition similarity between C-S-H gel and tobermorite [30,31,32,33,34]. The layered tobermorite model is composed of the arranged silica tetrahedrons dominated by the Dreierketten rule and a calcium layer [35]. Generally, tobermorite is categorized into 3 forms with spacings of 9 Å, 11 Å and 14 Å [36,37], respectively, according to the degree of hydration. Tobermorite 11 Å (T11) better reflects the nanocrystals and defective C-S-H crystals produced by silicate cement hydration [38], and it is unique in its central role in the family and its behavior during dehydration [39,40]. In this work, the tobermorite model with a space of 11 Å was employed for the following simulations, which has a chemical formula of Ca_4.5_Si_6_O_16_(OH)·5H_2_O (space group B11m). The lattice parameters are as follows: *a* = 6.73 Å, *b* = 7.37 Å, *c* = 22.68 Å, *α* = *β* = 90°, *γ* = 123.18° [41]. As shown in Figure 1, the relaxed crystal structure consists of interlayer calcium ions, calcium polyhedral layers, silicate tetrahedral chains and water molecules located in the interlayer spaces. Calcium ions can be classified into three types according to the oxygen coordination number and types. In detail, they are the Ca1 site (with 6 coordinated oxygen atoms contributed by the interlayered water and silicate tetrahedral chains), the Ca2 site (with 7 coordinated oxygen atoms contributed by the silicate tetrahedral chains) and the Ca3 site (with 7 coordinated oxygen atoms contributed by the interlayered water and silicate tetrahedral chains) see Figure 2a. Since the alkaline earth metal ions Mg^2+^, Ba^2+^ and Sr^2+^ have the same valence state and similar radius as Ca^2+^ [11], and the corresponding oxide configurations are the same as those of CaO, the introduction of doping elements at each of the three sites is considered to replace the Ca ion. The structures were fully optimized by the DFT calculations, and the elastic properties were calculated.

All the electronic structure calculations were based on the Perdew–Burke–Ernzerhof (PBE) generalized gradient approximation (GGA) exchange–correlation functional using a projector augmented-wave method [42,43,44]. For the plane-wave expansion, a 600 eV energy cutoff was set according to the convergence tests. The convergence criteria were 1.0 × 10^−5^ eV for the total energy change, and 0.01 eV/Å for the maximum allowed forces acting on each atom. The smearing value was set as 0.2 eV. The K-points were set to be 6 × 5 × 1 for the pristine and doped T11. DFT-D3, an approximate method developed by Grimme et al., was used to consider the van der Waals interactions’ contributions [45,46]. The Integrated Crystal Orbital Hamilton Population (ICOHP) analysis was applied to study the molecular orbital interactions of the specific atoms.

### 2.2. Properties Characterization

Firstly, the defect formation energy (*E_formation_*) is calculated according to $E_{formation}=E_{defect}-E_{tobermorite}-\sum_{i}n_{i}\mu_{i}$, where *E_defect_* and *E_tobermorite_* refer to the energy of doped and pristine structures. $n_{i}$ and $\mu_{i}$ refer to the number and the chemical potential of the doped atoms, respectively. Following this, elastic constants of the optimized pristine and doped tobermorite were calculated using the energy–strain method [47]. In detail, a small strain was applied on the equilibrate crystal cell first. Based on the system energy variation, the elastic stiffness tensor can be obtained by the second derivative of the total energy–strain curve. The elastic energy variation ($\Delta E(V,\{\varepsilon_{i}\})$) of the crystal can be described by

(1) $$\Delta E(V,\{\varepsilon_{i}\})=E(V,\{\varepsilon_{i}\})-E(V_{0},0)=\frac{V_{0}}{2}\sum_{i,j=1}^{6}C_{ij}\varepsilon_{j}\varepsilon_{i}$$

where $E(V,\{\varepsilon_{i}\})$ and $E(V_{0},0)$ represent the energy of the deformed and the equilibrated crystal with the volume of *V* and *V_0_*, respectively. $\varepsilon_{i}$ and $\varepsilon_{j}$ refer to the strain tensors of the corresponding crystal. The bulk modulus (*K*), shear modulus (*G*), Young’s modulus (*E*) and Poisson’s ratio (*v*) were evaluated based on the Voigt–Reuss–Hill (VRH) approximation [48,49], according to

(2) $$K_{VRH}=\frac{K_{V}+K_{R}}{2}$$

(3) $$G_{VRH}=\frac{G_{V}+G_{R}}{2}$$

(4) $$K_{V}=\frac{1}{9}[C_{11}+C_{22}+C_{33}+2(C_{12}+C_{13}+C_{23})]$$

(5) $$G_{V}=\frac{1}{15}[C_{11}+C_{22}+C_{33}-(C_{12}+C_{13}+C_{23})+3(C_{44}+C_{55}+C_{66})]$$

(6) $$K_{R}={\left[S_{11}+S_{22}+S_{33}+2(S_{12}+S_{13}+S_{23})\right]}^{-1}$$

(7) $$G_{R}=\frac{15}{4}{\left[S_{11}+S_{22}+S_{33}-(S_{12}+S_{13}+S_{23})+\frac{3}{4}(S_{44}+S_{55}+S_{66})\right]}^{-1}$$

(8) $$E_{VRH}={\left(\frac{1}{3K_{VRH}}+\frac{1}{9K_{VRH}}\right)}^{-1}$$

(9) $$v=\frac{3K_{VRH}-2G_{VRH}}{6K_{VRH}+2G_{VRH}}$$

where *S_ij_* are the matrix elements of the compliance tensor *S* = *C*^−1^.

These are the important formulas and methods used in this paper, followed by a detailed discussion of the effects of elemental doping on the structural and mechanical properties of tobermorite.

## 3. Results and Discussion

### 3.1. Formation and Structure of Doped Tobermorite

Table 1 illustrates the DFT-calculated crystal parameters of the pristine tobermorite, which align in general with the previous DFT results [50], and the experimental measurements (with an error within 1.5%). Compared with the experimental results, the DFT calculation slightly overestimates the lattice constants *a* and *b*, while the lattice constant *c* is slightly underestimated due to the interaction between the interlayer calcium and water molecules. As shown in Figure S1, the bond lengths of the Ca-O, Si-O and O-H bonds are mainly distributed in the range of 2.4~2.5 Å, 1.61~1.67 Å and 0.98~0.99 Å, respectively. Overall, the accuracy of the DFT calculations is acceptable.

Then, we investigate the formation energy of the doped tobermorite by examining three doping sites Ca1, Ca2 and Ca3 (Figure 2a). According to Figure 2b, the doping difficulty of the three elements: Mg > Ba > Sr. The average values of the *E_formation_* are ~1.14 eV, ~0.64 eV and 0.10 eV, respectively. For the same element specifically, the *E_formation_* of Ca1-doped sites is the smallest, which may be due to the relatively weak interaction between the interlayer calcium atoms and the bound water molecules. Previous studies have shown that intercalated cations are more easily substituted with external ions in the structure of tobermorite [39,51,52]. We believe that there should be a relationship between the differences in elemental doping difficulty and the structure, and for this reason, we analyze the changes in lattice parameters of doped tobermorite.

**Figure 3:**
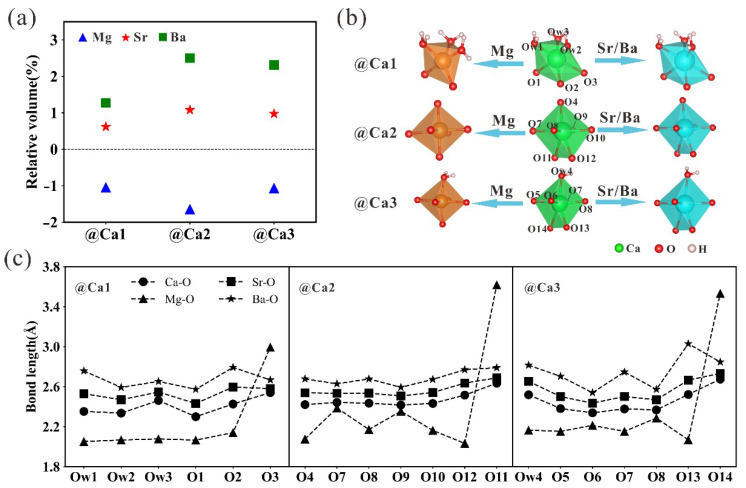
(**a**) The relative volume of tobermorite doped with different elements. Here, the relative volume is calculated from (*V* − *V*_0_)/*V*_0_ × 100%. (**b**) Relaxed x-oxygen (x = Ca, Mg, Sr, Ba) polyhedron structures. (**c**) Bond length information of x-oxygen polyhedrons.

The differences in the radii of dopant ions and calcium ions lead to changes in the volume of the cytosol of doped tobermorite. The lattice parameters of tobermorite after doping with different elements are given in Table S1. We compared previously reported computational data [50] and assessed the effect of elemental doping by comparing the cell lattice parameters before and after doping. The smaller cell parameters obtained from our calculations using DFT compared to previously reported calculated data for Mg-doped tobermorite are a result of the introduction of an empirically calculated dispersion-based correction and larger K-points values in order to better characterize the van der Waals forces in the tobermorite system. In addition, Mg substitution leads to an overall decrease in the lattice parameter of the tobermorite structure, whereas doping with Sr and Ba increases the lattice parameter. For clarity, the changes in the volume of cells are shown in Figure 3a. As it can be seen, the doping of Mg ions smaller than the Ca ionic radius decreased the cell volume in the range of about 1.04~1.64%, while the doping of Sr and Ba ions larger than the Ca ionic radius increased the cell volume by 0.62~1.08% and 1.27~2.51%, respectively. For the differential effects of the three doping sites on cell volume, the substitution at Ca2 site has the largest influence on the cell volume, followed by the substitution at the Ca3 site, and the substitution at the interlayer Ca1 site has a relatively small influence on the cell volume. For the same element, the difficulty of the substitution of Ca at different sites is positively correlated with the degree of effect on cell volume after substitution. The lattice volume is induced by local structural changes, and according to Figure 3b, the shapes of corresponding polyhedrons remain almost unchanged after the substitutions at different Ca sites by Sr and Ba. For the Mg-doped structure, large distortions occur both in the crystal structures and atomic coordination, with the oxygen coordination of Ca1, Ca2 and Ca3 sites reduced from 6, 7, 7 to 5, 6, 6. This local structural aberration due to changes in the coordination number may be responsible for the large *E_formation_* required for the substitution of Ca by Mg. The bond length of doped structure is shown in Figure 3c. The Mg-O bond length is the shortest compared with the Ca-O, Sr-O and Ba-O bonds. The average bond length is 2.16 Å, while the Ca-O, Sr-O and Ba-O bond lengths are 2.45 Å, 2.55 Å and 2.71 Å, respectively. These observations explain the smaller cell volume of the Mg-doped structure.

**Figure 4:**
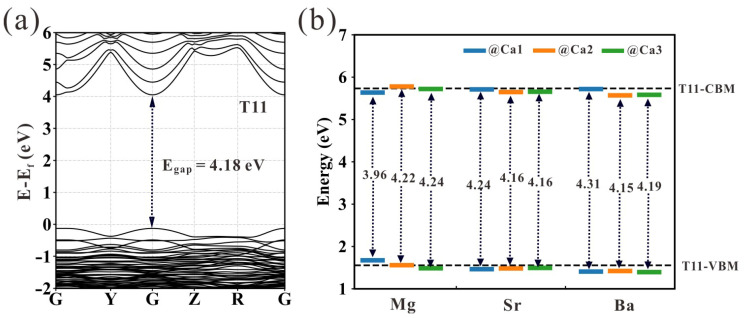
(**a**) The electronic band structure of pristine tobermorite and (**b**) the band gap of various doped tobermorites.

Based on the optimized geometry, the distribution of electronic orbits is obtained through electronic band structure calculations. The electronic band structure and the relative positions of the highest occupied orbit and the lowest unoccupied orbit (the size of the band gap (*E_gap_*)) are shown in Figure 4a,b, respectively. The change in chemical activity of tobermorite before and after doping can be quantified according to its *E_gap_*, which is about 4.18 eV for the pristine tobermorite, agree well with the reported values [28]. Compared with the pristine tobermorite, the *E_gap_* of the doped tobermorite varies from −5% to +1.5%. Among them, the band gaps of Mg@Ca2 and Ca3, Sr@Ca1, Ba@Ca1 and Ca3 sites become wider than 4.18 eV. The higher energy required for the transition of electrons from the top of the valence band to the bottom of the conduction band indicates reduced reactivity and enhanced stability. Meanwhile, for other doped structures, the stability of tobermorite shows a slight decrease.

### 3.2. Interaction between Doped Atoms and Surrounding Atoms

**Figure 5:**
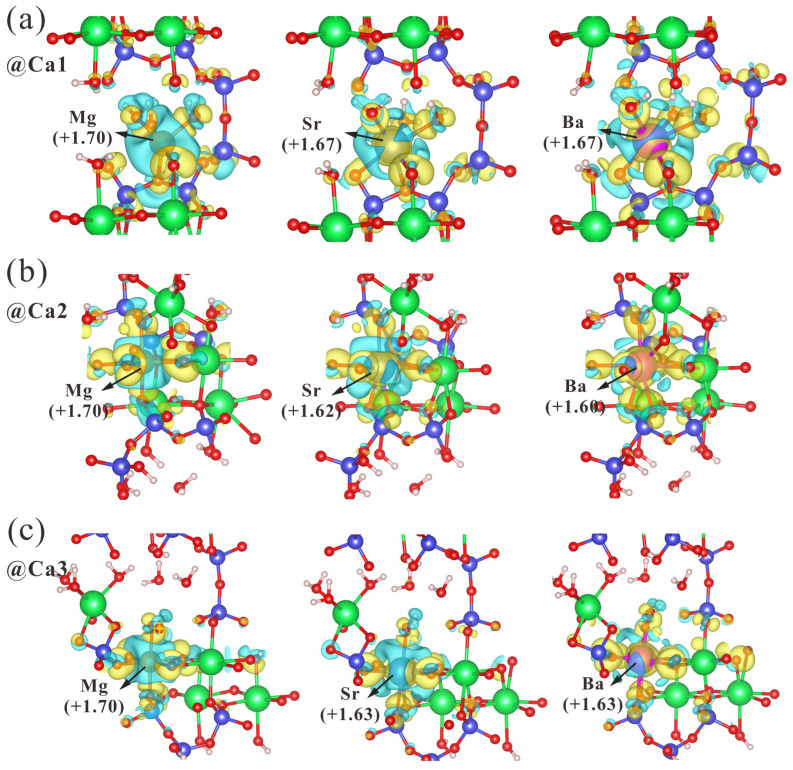
The valence charge and iso-surface of electron density difference of tobermorite doped with three elements at (**a**) Ca1, (**b**) Ca2 and (**c**) Ca3 sites. Sea blue: electron depletions; Yellow: electrons accumulations. (Isosurface value: 0.002 e/bohr^3^.).

**Figure 6:**
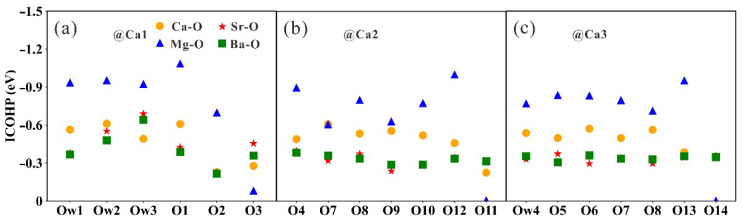
ICOHP analysis of Ca or doped atoms and surrounding oxygen atoms in (**a**) Ca1, (**b**) Ca2, and (**c)** Ca3 cites of tobermorite.

To exploit the interactions between the doped atoms and the surrounding atoms, electron density difference and valence charge analyses are conducted. The electron density difference shows electrons transferred from the doped atoms to the surrounding oxygen atoms, and the valence charge specifies the dissipation and aggregation of electrons, with Mg exhibiting a valence of +1.70 in tobermorite, losing more electrons than Sr and Ba, as illustrated in Figure 5. It can be found that there are differences in the mode of action of the dopant atoms at the three sites, with the dopant atom at the Ca2 site interacting only with the oxygen in the silicate chain. In contrast, the Ca1 and Ca3 sites interact with water molecules in addition to oxygen in the silicate chain. Moreover, the weak electron accumulation and dissipation around oxygen atoms in the cross-section of the electron density difference clearly explains the decrease in the number of oxygen coordination in Mg-doped tobermorite (See Figure S2). Furthermore, the Integrated Crystal Orbital Hamilton Population (ICOHP) analysis can shed light on the interatomic chemical bonding in doped tobermorite. Figure 6 shows that ICOHP between Mg-O at the three sites is more negative than Ca-O, Sr-O and Ba-O, with an average value of −0.836 eV, whereas the average ICOHP values of Ca-O, Sr-O and Ba-O are −0.479 eV, −0.367 eV and −0.357 eV, respectively. This indicates that Mg is tightly bound to the surrounding O, which is consistent with the change in bond length and electron transfer discussed above. In addition, the results of Rego [50] are in agreement with ours and explain the stronger Mg-O bond than Ca-O bond by electronegativity (χ_Mg_ = 1.30, χ_Ca_ = 1.0 [53]), although the electronic structure can provide a better proof. In detail, Figures S3 and S4 reveal the reason for the stronger interaction between doped Mg and surrounding O atoms. There are a large proportion of bonding states between Mg-O, mainly from the interaction between the *3s* orbital of Mg and the *2s* or *2p* orbital of O, whereas a smaller proportion of anti-bonding states between Mg-O is contributed by the Mg (*3s*)-O (*2p*) interactions. In addition, the main reason for the weak Sr-O and Ba-O interactions is that there are more anti-bonding components between Sr or Ba and O, which is formed by the interaction of *s*, *p* orbitals of doped atoms and *2p* orbitals of O.

### 3.3. The Mechanical Properties

**Figure 7:**
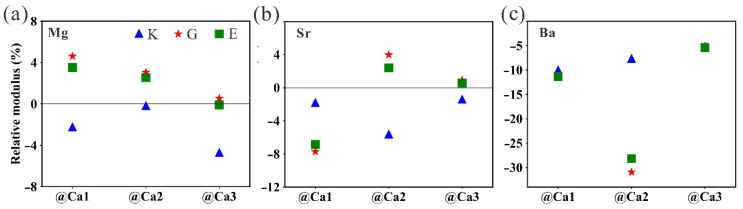
The relative changes in elastic modulus of tobermorite doped with Mg, Sr and Ba at (**a**) Ca1, (**b**) Ca2 and (**c**) Ca3 sites.

With the above understanding, we then explore the influence of elemental doping on the mechanical properties of tobermorite. The elastic coefficient of the pristine tobermorite is calculated in Table S2. The skeleton of tobermorite is composed of bisilicate chains with Si-O covalent bonds extending along the ab plane, and the intercalation will be connected between the two silicate chains by Si-O-Si covalent bonds, except for free water molecules and calcium ions. This structure implies that the tobermorite has similar stiffness in the three axial directions abc. C_11_ = 121.65 GPa, C_22_ = 135.20 GPa and C_33_ = 135.98 GPa corroborate this structural feature, which is consistent with reported studies [28,54]. Here, the approximate mechanical properties of the pristine and elemental doped tobermorite are compared in Table 2. It can be seen that the bulk modulus of pristine tobermorite is 69.50 GPa, which is very close to the experimental value (about 71.00 GPa) [55]. Comparing the two Ca/Si (as 0.75 versus 0.65) tobermorites, there is a slight difference between the calculated values in this paper and the overall modulus of tobermorillonite reported in the literature, which originates from the contribution of the interlayer calcium ions. The relative changes in elastic modulus of tobermorite at different doping elements are shown in Figure 7a–c, respectively. Overall, Mg doping enhances the Young’s modulus (average ~1.99%) and shear modulus (average ~2.74%) of tobermorite, with doping at the Ca1 site providing the greatest enhancement in modulus hardness. This change is in agreement with previous studies [50]. The effect of Sr on the modulus of tobermorite depends on the doping site, when replacing Ca2 and Ca3 may improve the Young’s modulus and shear modulus of tobermorite. Moreover, Ba doping softens the modulus stiffness of tobermorite at the cell scale.

**Figure 8:**
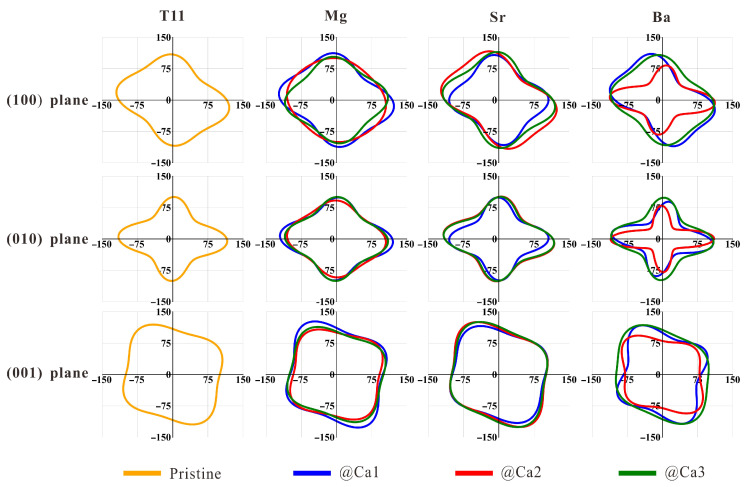
Projections of Young’s moduli of pristine and doped tobermorite on the (100), (010) and (001) crystal planes.

The projections of Young’s modulus in the (100), (010) and (001) planes for the pristine and doped tobermorite are given in Figure 8. The curve corresponding to an isotropic material is a standard circle. The greater the deviation of the curve from the circle, the more significant the anisotropy of the properties, a result that is detrimental to the durability of cementitious materials. The projection of the Young’s modulus of pristine tobermorillonite on the plane (010) is closest to the circle, and therefore, the Young’s modulus is almost the same on that plane. This is followed by the (100) plane, while there is a significant anisotropy in the (010) plane. Mg and Sr doping have little effect on the anisotropy of Young’s modulus of tobermorite in each plane. However, the anisotropy increases significantly when Ba is doped. Overall, considering the electronic structural stability of tobermorite and the elastic modulus change, these results suggest that Mg can be used as the preferred dopant to improve the mechanical properties of C-S-H.

## 4. Conclusions

In this study, effects of Mg, Sr and Ba doping on the structure and mechanical properties of tobermorite are systematically studied using DFT calculations. The formation of tobermorite doped with these three elements requires less energy. Of these, strontium and barium are more readily doped into tobermorite than magnesium. Specifically, all three elements are more suitable for doping into the Ca1 site of the interlayer, which has the least effect on the structure of topaz mullite. After replacing Ca with Sr and Ba, the lattice parameter of topaz mullite is enlarged, without significant changes in the local structure. However, the doping of Mg results in a large distortion of the structure and coordination of the oxygen polyhedra, which causes a decrease in the Mg-O bond length and lattice volume. This microstructural distortion in tobermorite is analyzed using the electronic structure, where the electrons of the doped atoms are transferred to the surrounding water molecules and oxygen in the silicate, where the Mg atoms will show a more positive valence due to the loss of more outer electrons. Moreover, the electron transfer clearly shows that the coordination number of Mg changes from 6 to 5 after substituting the interlayer Ca1, and from 7 to 6 after substituting Ca2 and Ca3. The electronic energy band structure shows that when Mg is doped at the Ca2 or Ca3 sites, and strontium or barium is doped at the Ca1 site, the Egap of the tobermorillonite will increase, and the chemical reactivity of the material will decrease and become more stable. Although Mg doping causes distortion in the structure of topaz mullite, ICOHP analysis shows that the Mg-O bond strength is higher, and elastic modulus calculations show that doping Mg at the Ca1 site in the interlayer improves the compressive and shear properties of topaz mullite to the greatest extent and has a small effect on the anisotropy of the Young’s modulus. It provides a promising strategy for C-S-H structure design.

## Figures and Tables

**Table 1:** Lattice parameters of the relaxation tobermorite Ca_4.5_Si_6_O_16_(OH)·5H_2_O.

	a (Å)	b (Å)	c (Å)	α (°)	β (°)	γ (°)
DFT [50]	6.834	7.411	22.707	89.87	90.45	122.89
This work	6.757	7.445	22.475	90.501	88.738	123.305
Exp. [41]	6.732	7.369	22.680	90.000	90.000	123.180
% diff.	0.377	1.029	−0.904	0.557	−1.402	0.101

**Table 2:** The bulk modulus *K* (GPa), shear modulus *G* (GPa), Young’s modulus *E* (GPa) and Poisson’s ratio (*v*) of tobermorite.

	*K_VHR_*	*G_VHR_*	*E_VHR_*	*v_VHR_*
This work (Ca/Si = 0.75)	69.50	37.44	95.22	0.27
Exp. (Ca/Si = 0.75)	71.00 ^a^	-	-	-
DFT (Ca/Si = 0.67)	68.08 ^b^, 66.12 ^c^, 77.19 ^d^	32.47 ^b^, 30.01 ^c^, 40.42 ^d^	78.20 ^c^, 103.25 ^d^	0.30 ^c^, 0.28 ^d^

^a^ Ref. [55]; ^b^ Ref. [56], ^c^ Ref. [54]; ^d^ Ref. [28].

## Data Availability

The data presented in this study are available on request from the corresponding author.

## Supplementary Materials

The following supporting information can be downloaded at: https://www.mdpi.com/article/10.3390/nano13162279/s1. Figure S1. Bond length distribution of (a) Ca-O, (b) Si-O and (c) O-H bonds of pristine T11. Figure S2. Two-dimensional cross-section of electron density difference of three elemental doped T11 at (a) Ca1, (b) Ca2 and (c) Ca3 sites. Blue: electron depletions; Red: electrons accumulations. Figure S3. Analysis of the orbital interaction between (a) Ca1-O, (b) Ca2-O and (c) Ca3-O in pristine T11. Figure S4. Analysis of the orbital interaction between doped atoms and oxygen atoms in Mg, Sr, Ba doped T11. (a–c) Mg-doped T11 with different doping sites, (d–f) Sr-doped T11 with different doping sites, and (g–i) Ba-doped T11 with different doping sites. Table S1. Lattice parameters and angles of Mg-, Sr-, and Ba-doped T11 and relative change from pristine T11. Table S2. Calculated and simulated elastic constants and other simulated values of pristine T11.
