# Supplementary material for: Structural and Mechanical Properties of Doped Tobermorite
Source: Nanomaterials (Basel). 2023 Aug 8;13(16):2279. doi: 10.3390/nano13162279 (PMC10459530; doi:10.3390/nano13162279)
Supplement: Supplementary file 1 [file nanomaterials-13-02279-s001.zip › nanomaterials-2497138-supplementary.pdf]

Supporting information

Structural and Mechanical Properties of Doped Tobermorite

Xiaopeng Li [1], Hongping Zhang [1,2,*], Haifei Zhan [3,4] and Youhong Tang [5,*]

1 School of Materials and Chemistry, Southwest University of Science and Technology, Mianyang 621010, China; lxp5202021@163.com
2 School of Mechanical Engineering, Institute for Advanced Study, Chengdu University, Chengdu 610106, China
3 College of Civil Engineering and Architecture, Zhejiang University, Hangzhou 310058, China; zhan_haifei@zju.edu.cn
4 School of Mechanical, Medical and Process Engineering, Queensland University of Technology (QUT), Brisbane 4001, Australia
5 Institute for NanoScale Science and Technology, College of Science and Engineering, Flinders University, Adelaide 5042, Australia
* Correspondence: zhp1006@126.com (H.Z.); youhong.tang@flinders.edu.au (Y.T.); Tel.: +86-816-6089009 (H.Z.); +61-8-82012138 (Y.T.)

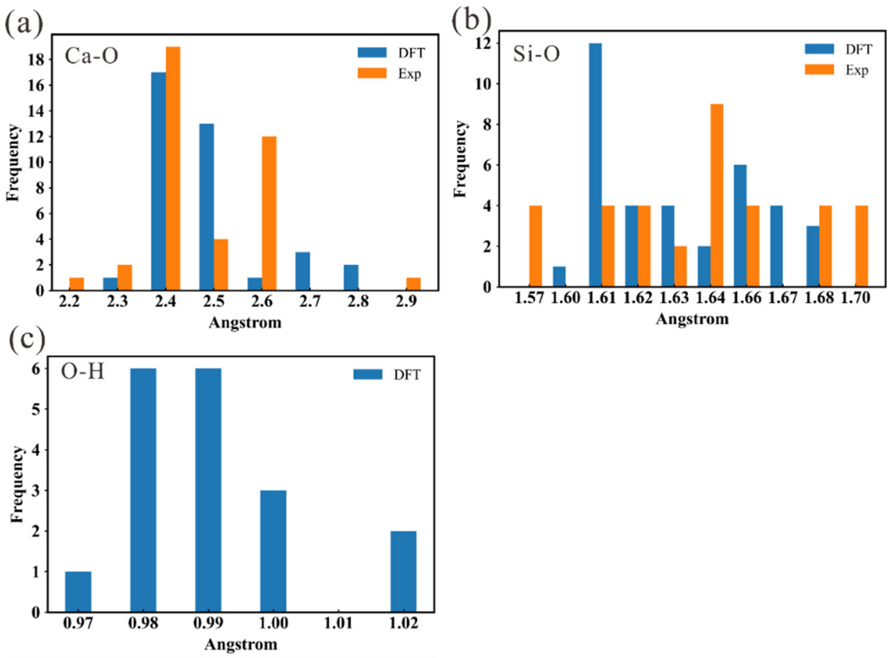

Figure S1. Bond length distribution of (a) Ca-O, (b) Si-O and (c) O-H bonds of pristine T11.

Citation: Li, X.; Zhang, H.; Zhan, H.; Tang, Y. Structural and Mechanical Properties of Doped Tobermorite. *Nanomaterials* **2023**, *13*, 2279. https://doi.org/10.3390/nano13162279

Academic Editor: Maciej Sitarz

Received: 26 June 2023
Revised: 29 July 2023
Accepted: 2 August 2023
Published: 8 August 2023

Copyright: © 2023 by the authors. Licensee MDPI, Basel, Switzerland. This article is an open access article distributed under the terms and conditions of the Creative Commons Attribution (CC BY) license (https://creativecommons.org/licenses/by/4.0/).

Table S1. Lattice parameters and angles of Mg-, Sr-, and Ba-doped T11 and relative change from pristine T11.

		a (Å)	b (Å)	c (Å)	α (°)	β (°)	γ (°)
Mg@Ca1	DFT [1]	6.796	7.485	22.572	89.64	90.18	123.164
	This work	6.730	7.439	22.397	90.373	88.889	123.489
	Relative change (%)	-0.41	-0.08	-0.35	-0.14	0.17	0.15
Mg@Ca2	-	6.753	7.411	22.224	90.823	89.926	123.323
	Relative change (%)	-0.065	-0.454	-1.117	0.355465	1.339	0.0147
Mg@Ca3	-	6.716	7.367	22.499	89.734	87.242	122.726
	Relative change (%)	-0.612	-1.045	0.106	-0.848	-1.686	-0.469
Sr@Ca1	-	6.776	7.451	22.498	90.647	88.812	123.171
	Relative change (%)	0.276	0.083	0.102	0.161	0.0835	-0.109
Sr@Ca2	-	6.791	7.460	22.542	90.525	88.833	123.249
	Relative change (%)	0.498	0.204	0.298	0.026	0.107	-0.045
Sr@Ca3	-	6.788	7.478	22.481	90.481	88.901	123.276
	Relative change (%)	0.453	0.446	0.026	-0.022	0.184	-0.023
Ba@Ca1	-	6.797	7.461	22.511	90.821	88.880	123.049
	Relative change (%)	0.586	0.218	0.160	0.353	0.160	-0.208
Ba@Ca2	-	6.833	7.485	22.625	88.848	90.551	123.169
	Relative change (%)	1.119	0.540	0.667	-1.827	2.043	-0.110
Ba@Ca3	-	6.826	7.518	22.465	90.517	89.811	123.022
	Relative change (%)	1.016	0.983	-0.045	0.017	1.209	-0.229

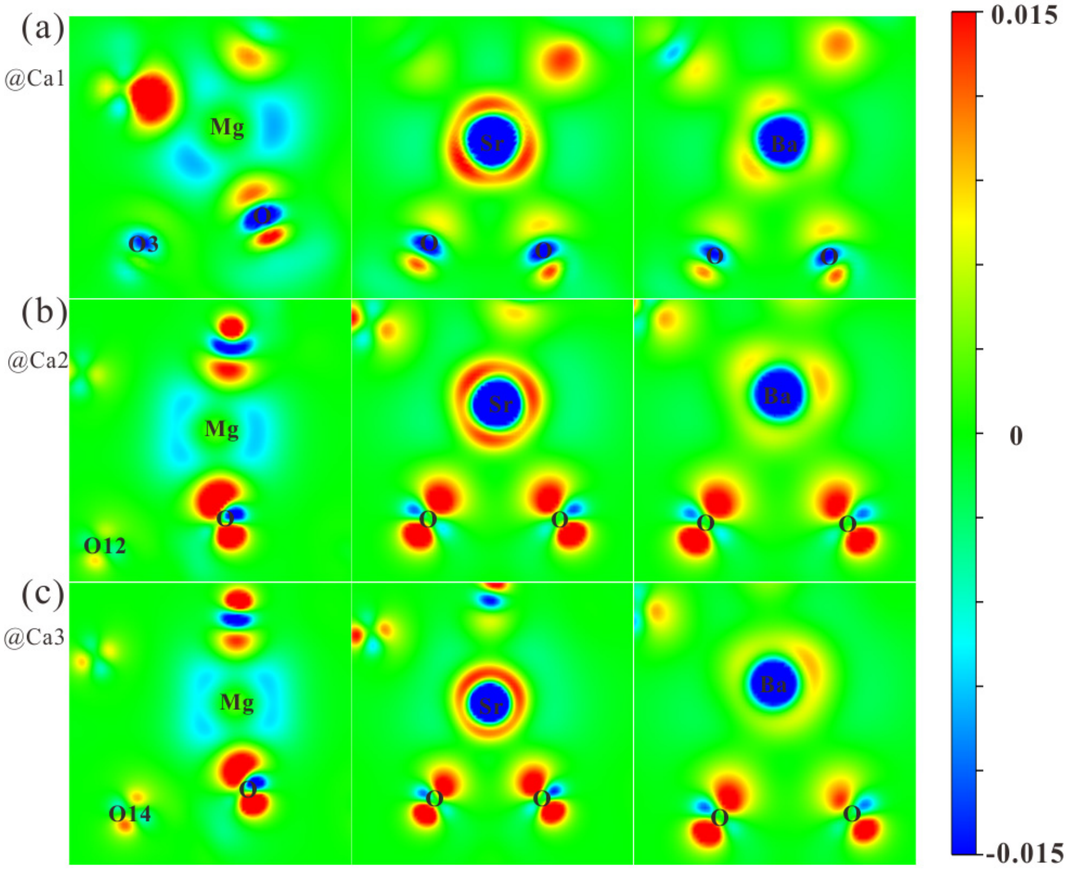

Figure S2. Two-dimensional cross-section of electron density difference of three elemental doped T11 at (a) Ca1, (b) Ca2 and (c) Ca3 sites. Blue: electron depletions; Red: electrons accumulations.

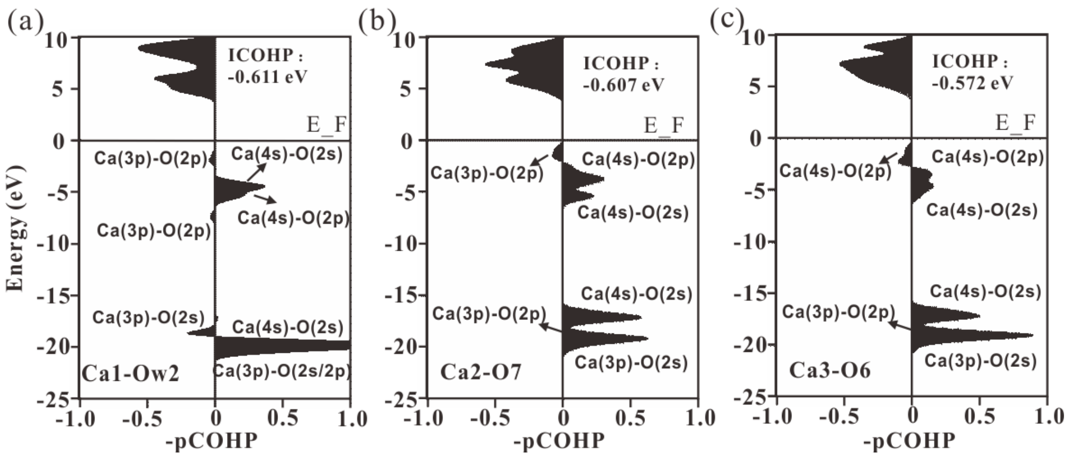

Figure S3. Analysis of the orbital interaction between (a) Ca1-O, (b) Ca2-O and (c) Ca3-O in pristine T11

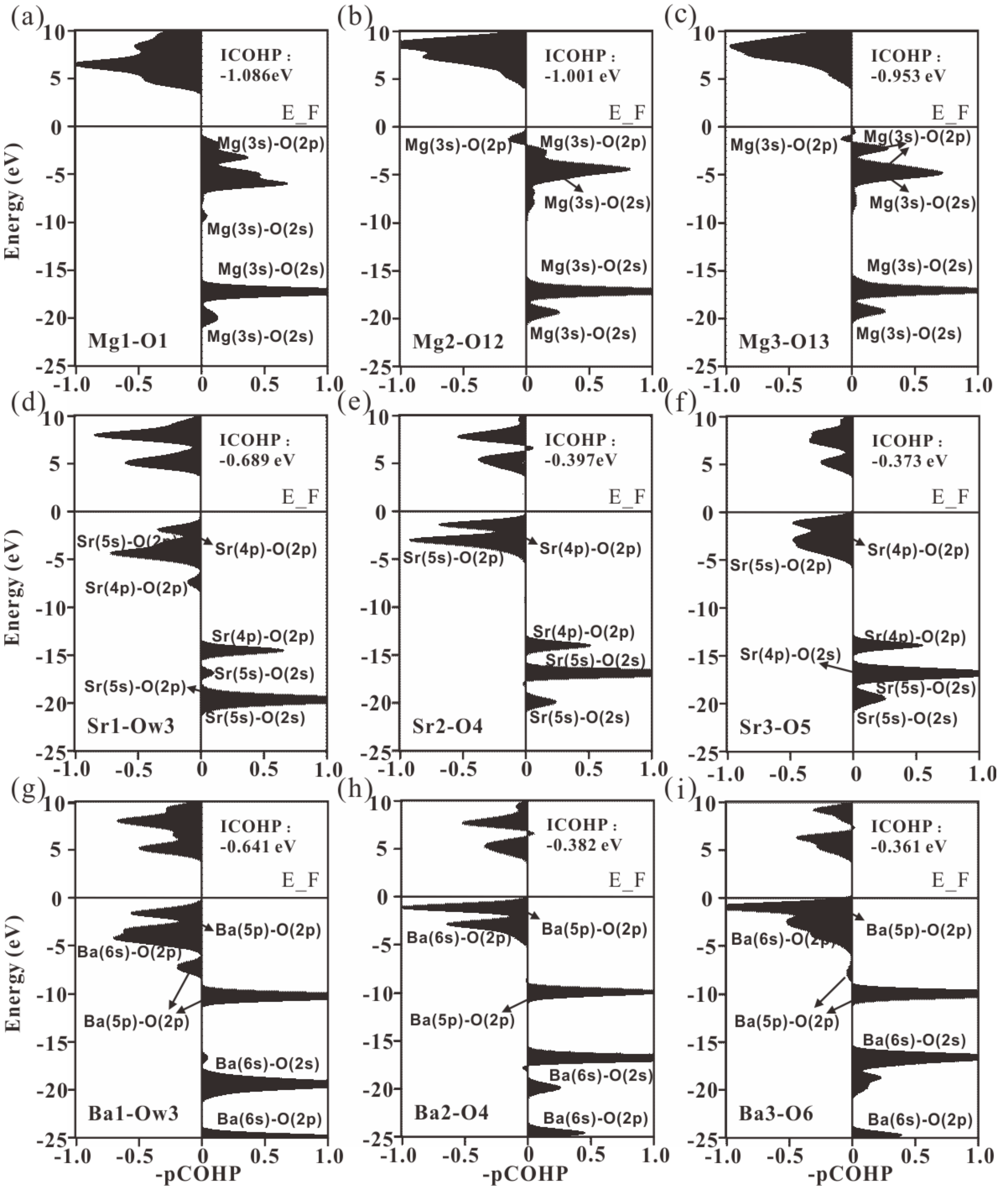

Figure S4. Analysis of the orbital interaction between doped atoms and oxygen atoms in Mg, Sr, Ba doped T11. (a) - (c) Mg-doped T11 with different doping sites, (d) - (f) Sr-doped T11 with different doping sites, and (g) - (i) Ba-doped T11 with different doping sites.

Table S2. Calculated and simulated elastic constants and other simulated values of pristine T11.

	C11	C12	C13	C22	C23	C33	C44	C55	C66
This work	121.65	47.98	34.89	135.20	39.31	135.98	36.25	24.88	50.02
DFT	115.73 [b],	47.67 [b],	35.04 [b].	131.26 [b],	42.12 [b],	127.62 [b],	26.53 [b],	22.26 [b],	46.15 [b],
(Ca/Si = 0.67)	112.51 [c],	47.68 [c],	32.54 [c],	128.89 [c],	36.25 [c],	141.17 [c],	25.98 [c],	17.20 [c],	46.85 [c],
	137.34 [d]	52.89 [d]	36.47 [d]	145.41 [d]	49.66 [d]	130.30 [d]	38.91 [d]	28.23 [d]	48.99 [d]

[b] Ref. [2]
[c] Ref. [3]
[d] Ref. [4]

References

1. Rego, J.S.; Miranda, C.R. and de Koning, M. Effects of $Ca^{2+} \rightarrow Mg^{2+}$ substitution on the properties of cementitious tobermorite. Physical Review Materials 2022, **6**, 063604.
2. Kobayashi, K.; Nakamura, H.; Yamaguchi, A.; Itakura, M.; Machida, M. and Okumura, M. Machine learning potentials for tobermorite minerals. Computational Materials Science 2021, **188**, 110173.
3. Zhou, Y.; Zheng, H.; Li, W.; Ma, T. and Miao, C. A deep learning potential applied in tobermorite phases and extended to calcium silicate hydrates. Cem. Concr. Res. 2022, **152**, 106685.
4. Dharmawardhana, C.C.; Misra, A.; Aryal, S.; Rulis, P. and Ching, W.Y. Role of interatomic bonding in the mechanical anisotropy and interlayer cohesion of CSH crystals. Cem. Concr. Res. 2013, **52**, 123-130.
